# Exploration of Tilmicosin Cardiotoxicity in Rats and the Protecting Role of the *Rhodiola rosea* Extract: Potential Roles of Cytokines, Antioxidant, Apoptotic, and Anti-Fibrotic Pathways

**DOI:** 10.3390/toxics11100857

**Published:** 2023-10-13

**Authors:** Salwa A. Elgendy, Mohamed Mohamed Soliman, Heba I. Ghamry, Mustafa Shukry, Lina Abdelhady Mohammed, Hend Elsayed Nasr, Badriyah S. Alotaibi, Ibrahim Jafri, Samy Sayed, Amira Osman, Heba A. Elnoury

**Affiliations:** 1 Department of Pharmacology, Faculty of Medicine, Benha University, Benha 13511, Egypt; 2 Department of Clinical Laboratory Sciences, Turabah University College, Taif University, P.O. Box 11099, Taif 21944, Saudi Arabia; mmsoliman@tu.edu.sa; 3 Biochemistry Department, Faculty of Veterinary Medicine, Benha University, Toukh 13736, Egypt; 4 Nutrition and Food Science, Department of Home Economics, Faculty of Home Economics, King Khalid University, P.O. Box 960, Abha 61421, Saudi Arabia; hgmry@kku.edu.sa; 5 Department of Physiology, Faculty of Veterinary Medicine, Kafrelsheikh University, Kafrelsheikh 33516, Egypt; 6 Department of Medical Biochemistry and Molecular Biology, Faculty of Medicine, Benha University, Benha 13511, Egypthend.mosalm@fmed.bu.edu.eg (H.E.N.); 7 Department of Pharmaceutical Sciences, College of Pharmacy, Princess Nourah bint Abdulrahman University, P.O. Box 84428, Riyadh 11671, Saudi Arabia; 8 Department of Biotechnology, College of Science, Taif University, P.O. Box 11099, Taif 21944, Saudi Arabia; 9 Department of Economic Entomology and Pesticides, Faculty of Agriculture, Cairo University, Giza 12613, Egypt; samy_mahmoud@hotmail.com; 10 Department of Science and Technology, University College-Ranyah, Taif University, P.O. Box 11099, Taif 21944, Saudi Arabia; 11 Department of Basic Medical and Dental Sciences, Faculty of Dentistry, Zarqa University, Zarqa 13110, Jordan; mero.osman@med.kfs.edu.eg; 12 Department of Histology and Cell Biology, Faculty of Medicine, Kafrelsheikh University, Kafrelsheikh 33516, Egypt

**Keywords:** cardiomyopathy, tilmicosin, *Rhodiola rosea*, antioxidants, oxidative stress, apoptosis

## Abstract

Tilmicosin (TIL) is a common macrolide antibiotic in veterinary medicine. High doses of TIL can have adverse cardiovascular effects. This study examined the effects of *Rhodiola rosea* (RHO) that have anti-inflammatory, antioxidant, and anti-fibrotic effects on tilmicosin (TIL)-induced cardiac injury targeting anti-inflammatory, antioxidant, apoptotic, and anti-apoptotic signaling pathways with anti-fibrotic outcomes. Thirty-six male Wistar albino rats were randomly divided into groups of six rats each. Rats received saline as a negative control, CARV 1 mL orally (10 mg/kg BW), and RHO 1 mL orally at 400 mg/kg BW daily for 12 consecutive days. The TIL group once received a single subcutaneous injection (SC) dose of TIL (75 mg/kg BW) on the sixth day of the experiment to induce cardiac damage. The standard group (CARV + TIL) received CARV daily for 12 consecutive days with a single TIL SC injection 1 h after CARV administration only on the sixth day of study and continued for another six successive days on CARV. The protective group (RHO + TIL) received RHO daily for the same period as in CARV + TIL-treated rats and with the dosage mentioned before. Serum was extracted at the time of the rat’s scarification at 13 days of study and examined for biochemical assessments in serum lactate dehydrogenase (LDH), cardiac troponin I (cTI), and creatine phosphokinase (CK-MB). Protein carbonyl (PC) contents, malondialdehyde (MDA), and total antioxidant capacity (TAC) in cardiac homogenate were used to measure these oxidative stress markers. Quantitative RT-PCR was used to express interferon-gamma (INF-γ), cyclooxygenase-2 (COX-2), OGG1, BAX, caspase-3, B-cell lymphoma-2 (Bcl-2), and superoxide dismutase (SOD) genes in cardiac tissues, which are correlated with inflammation, antioxidants, and apoptosis. Alpha-smooth muscle actin (α-SMA), calmodulin (CaMKII), and other genes associated with Ca^2+^ hemostasis and fibrosis were examined using IHC analysis in cardiac cells (myocardium). TIL administration significantly increased the examined cardiac markers, LDH, cTI, and CK-MB. TIL administration also increased ROS, PC, and MDA while decreasing antioxidant activities (TAC and SOD mRNA) in cardiac tissues. Serum inflammatory cytokines and genes of inflammatory markers, DNA damage (INF-γ, COX-2), and apoptotic genes (caspase-3 and BAX) were upregulated with downregulation of the anti-apoptotic gene Bcl-2 as well as the DNA repair OGG1 in cardiac tissues. Furthermore, CaMKII and α-SMA genes were upregulated at cellular levels using cardiac tissue IHC analysis. On the contrary, pretreatment with RHO and CARV alone significantly decreased the cardiac injury markers induced by TIL, inflammatory and anti-inflammatory cytokines, and tissue oxidative-antioxidant parameters. INF-γ, COX-2, OGG1, BAX, and caspase-3 mRNA were downregulated, as observed by real-time PCR, while SOD and Bcl-2 mRNA were upregulated. Furthermore, the CaMKII and α-SMA genes’ immune reactivities were significantly decreased in the RHO-pretreated rats.

## 1. Introduction

Tilmicosin (TIL) is a popular semi-synthetic macrolide antibiotic in veterinary medicine. It binds to the 50S ribosomal subunit and prevents protein synthesis in bacteria. This compound has 16 atoms. Treatment and prevention of pneumonia caused by *P. multocida*, *Actinobacillus pleuropneumonia*, *Pasteurella haemolytica*, *Streptococcus*, *Staphylococcus*, and *Mycoplasma* are among its approved uses in cattle, sheep, and pigs [1]. In addition, it is used in ruminant animals to avoid and treat mastitis [2]. Low inhibitory concentrations, a high distribution volume, and a long elimination half-life contribute to TIL’s effectiveness [3]. TIL has been shown to have a wide range of negative consequences in research, some of which are cardiotoxic [4,5], and the heart is the main target for Tilmicosin action [6].

Several animal studies have shown that high doses of TIL have adverse inotropic and positive chronotropic cardiovascular effects, including ventricular function impairment, abrupt heart failure, and ECG alterations [7]. Many previous studies have not clearly described how TIL induces its cardiotoxicity effect. However, TIL may induce cardiotoxicity through increased production of reactive oxygen species (ROS) or affect the oxidative state of the heart tissues [8] through protein, DNA, and lipid damage, leading to apoptosis [9].

Calmodulin (CaM) is a tiny protein in the cytoplasm with two globular domains that serve as Ca^2+^ binding sites [10]. Muscle contraction in both cardiac and skeletal muscles results from the cytoplasmic release of Ca^2+^ ions through ryanodine receptor calcium release channels (RyRs); RyR2 is prominent in cardiac muscle, while RyR1 is found at high levels in skeletal muscle. Calmodulin in the muscles of the skeleton and the heart is one of several related proteins with RyRs, which are large ion channels made up of four peptide subunits, four tiny 12 kDa FK506 binding proteins (FKBP), and other proteins [11].

Carvedilol is an anti-inflammatory, anti-hypertensive, and anti-ischemic medication for the cardiovascular system with multiple approved uses. In individuals with congestive heart failure, it boosts myocardial function, prolongs life, and reduces mortality [12]. Catecholamines are harmful to the heart. However, this compound can prevent its effects [13]. Carvedilol has recently been shown to reduce the severity of ischemia and reperfusion injury through its antioxidant action, calcium antagonism, anti-arrhythmia, anti-apoptosis, and neutrophil infiltration inhibition in human and animal investigations. In addition, carvedilol can switch myocardial substrates from free fatty acids to glucose oxidation [14]. As a result, there is a greater need to discover new antioxidants derived from natural sources that are efficacious, bio-efficient, and non-toxic for use as safe therapeutic agents [15].

*Rhodiola rosea* (RHO) is a valuable phytomedicine cultivated in Tibet and China’s cold and high altitudes. A member of the Crassulaceae family of plants, “golden root” or “roseroot” is used as a botanical adaptogen all over the world [16]. RHO has been shown to enhance cardiovascular and cerebral blood flow systems. Different physiologically active chemicals found in RHO may have other effects [17]. The glycoside molecule rhodioloside (salidroside) and the class of rosavins primarily found in plant rhizomes, including rosavin, rosarin, and rosin [18], have shown medicinal properties. Multiple investigations have identified salidroside as RHO’s most dynamic ingredient [19]. RHO has a complex chemical structure, and its pharmacological effects and therapeutic efficacy for treating a wide range of cardiovascular illnesses are also highly variable among its numerous chemical components [20].

Multiple biological functions, including immune regulation, antioxidants, and cancer cell proliferation inhibition, have been attributed to *Rhodiola rosea* L. preparations by pharmacological researchers in both clinical practice and experimental studies [16]. It has been found that RHO extract has anti-inflammatory properties and protects muscular tissue during exercise [21]. It also has various protective products such as anti-cancer, anti-aging, anti-diabetic, neuroprotective, and cardioprotective effects [16]. The impact of stress on rats’ eating, exercise, and reproductive cycles was mitigated by a compound called RHO [22].

Therefore, the current study aimed to outline the protective impacts of RHO that might be involved in ameliorating cardiotoxicity induced by TIL injection. Various biochemical, molecular, and cellular signaling pathways were confirmed to regulate apoptosis, oxidative stress, fibrosis, inflammation, and anti-apoptotic markers.

## 2. Materials and Methods

### 2.1. Drugs and Chemicals

A commercial preparation for tilmicosin (Micotil^®^ 300 solution; AH0230) was obtained from (Elanco animal health, Macquarie Park, NSW, Australia)—Dilatrend (25 mg carvedilol, Roche SpA, Segrate MI, Italy). *Rhodiola rosea* extract was obtained from Puritan’s Pride^®^ Company (Puritan’s Pride, Oakdale, NY, USA) as capsules containing 250 mg of Rhodiola root extract. All additional chemicals and reagents used in this investigation were bought from (Sigma-Aldrich Co., St. Louis, MO, USA) and were of the highest commercial quality.

### 2.2. Gas Chromatography–Mass Spectrometry (GC-MS) Analysis of Rhodiola rosea Extract

The chemical composition analysis of *Rhodiola rosea* extract was conducted using a Trace GC-TSQ mass spectrometer (Thermo Scientific, Austin, TX, USA) equipped with a direct capillary column TG–5MS (30 m × 0.25 mm × 0.25 µm film thickness). Initially, the column oven temperature was maintained at 50 °C and then gradually increased at a rate of 5 °C per minute until it reached 250 °C, where it was held for 2 min. Subsequently, it was further increased to the final temperature of 300 °C at 30 °C per minute and held for 2 min. The injector and MS transfer line temperatures were maintained at 270 °C and 260 °C, respectively. Helium gas was the carrier gas at a constant 1 mL/min flow rate. A solvent delay of 4 min was incorporated, and diluted samples of 1 µL were automatically injected using the Autosampler AS1300 (Thermo Scientific, Austin, TX, USA) in the split mode. Electron ionization (EI) mass spectra were obtained using an ionization voltage of 70 eV, covering the *m*/*z* range of 50–650 in full scan mode. The ion source temperature was set at 200 °C. The components present in the extract were identified by comparing their mass spectra with those available in the WILEY 09 and NIST 14 mass spectral databases [23].

### 2.3. Animal Grouping and Treatment

Thirty-six male Wistar rats (180–200 gm) and two months’ age were purchased from an animal breeding farm (Helwan-Cairo, Egypt). Rats were housed in stainless steel cages, monitored for two weeks before the experiment to ensure normal growth and behavior, and kept in a controlled environment with a temperature of 25. 5 °C, a humidity of 55.5%, a 12 h light/dark cycle, and free access to standard rat feed (El-Nasr Co., Cairo, Egypt). Animals were handled manually to ensure complete acclimatization. After acclimatization, rats were distributed randomly into groups of six rats per group. Group I (normal) rats were intubated with 1 mL of distilled water in the stomach tube for 12 consecutive days. Group II (Rhodiola-treated) (RHO): rats received RHO 1 mL orally (400 mg/kg BW daily) [24] for 12 consecutive days. Group III (Carvedilol-treated) (CARV) rats received CARV 1 mL orally at a dose of 10 mg/kg BW [25,26,27] for 12 consecutive days. Group IV (Tilmicosin-treated; TIL): rats received a single subcutaneous injection (SC) at a dose of 75 mg/kg BW [4,28] on the sixth day of the experiment. Group V (CARV + TIL): rats obtained CARV for 12 consecutive days with a single TIL S/C injection 1 h after CARV administration only on the sixth day of study and continued for another six successive days on CARV with the exact dosage mentioned before. Group VI (RHO + TIL): rats obtained RHO for 12 consecutive days with a single TIL S/C injection 1 h after RHO administration on the sixth day of study only and continued for another six successive days on RHO with the exact dosage mentioned before. Each control, RHO, and CARV rat received a single subcutaneous (S/C) injection of physiological saline (NaCl 0.9%). On day 6, the other three groups only received one S/C injection of TIL. The LD50 of dry R. rosea extract for rats is 3.36 g/kg [29,30], while the oral LD50 doses of carvedilol for mice and rats are over 8000 mg/kg [31].

On the final day of the experiment, the animals had free access to water but were otherwise fasted for 12 h. After that, we used cervical dislocation to end the rats’ lives and collected blood samples by puncturing their hearts. Serum samples obtained after centrifuging the collected blood at 3000 rpm for 10 min were stored at 20 °C for further analysis. Next, the heart was taken, rinsed with saline, dried, weighed, and dissected. The heart tissue was divided into three equal longitudinal parts. The heart tissue was frozen and stored at 80 °C in a flash. The frozen materials were mixed in a cold phosphate-buffered saline solution (0.1 M, pH 7.4) in a homogenizer fitted with a Teflon pestle and centrifuged at 5000× *g* for 15 min at 4 °C. For the chemical analysis, aliquots of the supernatant were frozen at 80 °C. Other cardiac tissues were promptly preserved in a neutral buffered formalin 10% solution for histopathological and immunohistochemical analysis [32].

### 2.4. Assessment of Body Weight and Cardiac Injury Biomarkers

Each rat’s total body weight was recorded after the completion of the experiment. As per package directions, we estimated serum LDH and CK-MB concentrations. Serum cTI was also evaluated with an ELISA Plate Reader (Bio-Rad, Hercules, CA, USA) and an ELISA kit specific for the measurement of cTnI. MyBiosource, Inc. (San Diego, CA, USA) kindly provided rat cTnI level ELISA kits. Tsung’s method [33] was used to measure creatine kinase-MB (CK-MB), and Lum and Gambino’s method [34] was used to calculate LDH.

### 2.5. Inflammatory and Anti-Inflammatory Cytokine Assessments

Serum concentrations of IL-1 and TNF-alpha were measured using specific ELISA kits (ab255730 and ab46070, respectively) and spectrophotometric analysis according to the manufacturer’s instructions. Rat IL-10 ELISA Kit (ab100765), a commercially available kit for measuring IL-10, was purchased from (abcam, Cambridge, MA, USA). The ELISA reader’s data was approximated and evaluated according to the kit’s instructions.

### 2.6. Oxidative Stress Biomarker (ROS, PC, MDA, TAC, 8-OHdG, and OGG1) Assessments in Cardiac Tissue Homogenate

The concentration of reactive oxygen species (ROS) in rat heart tissue was determined using an ELISA kit (MBS164653; MyBioSource Co., San Diego, CA, USA) following the manufacturer’s instructions. Cayman’s Chemical Co., located at 1000 Ellsworth Road, Ann Arbor, MI, USA, produced a colorimetric assay pack (no. 10005020) to measure protein carbonyl (PC) levels. According to Ohkawa et al.’s findings [35], malondialdehyde (MDA) levels were determined by a colorimetric test. The total antioxidant capacity (TAC) was measured using a kit from Biodiagnostic Co., Dokki, Giza, Egypt (catalog no. TA 2513; Egypt).

The My BioSource rat ELISA kits were used to estimate the levels of 8-OHdG (MBS269902) in the heart, following the manufacturer’s instructions. To quantify the content of OGG1, a commercially available 96-well ELISA kit (CSB-EL016313RA, Cusabio Biotech Co., Ltd., College Park, MD, USA) was used. The ELISA assays were performed following the manufacturer’s instructions. In brief, brain tissue was homogenized in a lysis buffer solution. The samples and standards were added to 96-well plates and incubated at 37 °C for 90 min. After washing three times, a biotinylated secondary antibody was added and incubated for 1 h at 37 °C. Following two more washes, an enzyme conjugate liquid was added and incubated for 30 min at 37 °C. After five washes, a color reagent was added to each well and incubated at 37 °C for 30 min in the dark. The absorbance was measured at 450 nm after adding the stop solution.

### 2.7. qRT-PCR Analysis

The quantitative expression of genes in cardiac tissue was measured and expressed using quantitative real-time PCR. About 100 ng of cardiac tissue was briefly used with Qiazol reagents to extract RNA. cDNA was synthesized with a Multi-Scribe RT-enzyme kit. 7500 Real-Time PCR equipment(Applied Biosystems, Waltham, MA, USA) was used to run qRT-PCR reactions using SYBR Green staining PCR Master Mix. The 2CT technique was used to quantify these genes. The examined genes were normalized to the housekeeping gene actin. The relative cycle threshold (CT) values were used to study the variations in gene expression [36]. Table 1 displays the genes that were analyzed.

### 2.8. Cardiac Histopathology and Immunohistochemistry

All cardiac tissue (6 rats per group) was removed, fixed in 10% neutral buffered formalin for 72 h, processed into paraffin blocks, and then sectioned longitudinally at 5 m. The sections were stained with hematoxylin [37]. The previously published free-floating technique was used for immunohistochemistry [38]. Anti-calmodulin ab2860 polyclonal antibody, Abcam (Cambridge, UK), was used as the primary antibody for CaMKII, Ca^2+^/calmodulin-dependent protein kinase II. Brown cytoplasmic deposits were detected by immunohistochemistry employing monoclonal antibodies directed against alpha-smooth muscle actin (α-SMA) (Lab vision neo markers USA, Catalog No. 1-GE002-07), a sign of active myofibroblasts [39].

### 2.9. Western Blotting

Tissues were lysed using RIPA lysis buffer (Solarbio, Beijing, China). Protein concentrations were determined using an improved BCA protein assay kit (Beyotime, Shanghai, China). After centrifugation, equal amounts of protein (50 g) were loaded onto a 10% SDS-polyacrylamide gel and transferred to polyvinylidene fluoride membranes (Merck Millipore, Boston, MA, USA). The membranes were blocked in 5% skimmed milk in TBS and incubated overnight at 4 °C with primary antibodies against OGG1 (1:400; Abcam) and β-actin (1:1000; Santa Cruz Biotechnology, Inc., Dallas, TX, USA). Subsequently, the membranes were washed and incubated with horseradish peroxidase-conjugated secondary antibody at 37 °C for 2 h. After further washes, the bands were quantified using (Image-J software 6.1; ImageJ 1.45 s freeware, National Institutes of Health, Rockville, MD, USA), with the optical densities normalized to β-actin.

### 2.10. Statistical Analysis

SPSS (SPSS Inc., Chicago, IL, USA), version 26 for Windows, tabulated and analyzed the data. The data were presented as a mean + SD. The Shapiro–Wilks test was used to check if the data were normally distributed (t *p* > 0.05). One-way analysis of variance (ANOVA) was used to look for deviations in normally distributed variables. After a statistically significant analysis of variance (ANOVA), we performed post hoc Tuckey HSD multiple comparisons with Bonferroni testing to identify statistically significant pairs. In this study, *p* < 0.05 was indicated as the threshold for statistical significance.

## 3. Results

### 3.1. Chromatographic Pattern of GC-MS Spectral Analysis of Rhodiola rosea Extract

**Figure 1:**
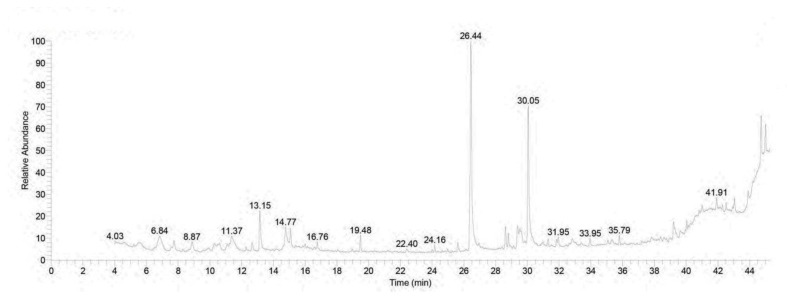
Pattern of GC-MS spectral analysis.

Table 2 and Figure 1 present the detected compounds and their chemical formulas, peak areas, retention times, and molecular weights. A total of 32 peaks were observed, including n-Hexadecanoic acid (26.47%), á-Sitosterol (5.39%), Stigmast-5-EN-3-ol, (3á,24S) (5.39%), 2-Propenoic acid, 3-phenyl-, methyl ester (5.11%), Linalool (3.62%), 1,6-Octadien-3-ol, 3,7-dimethyl (3.62%), 7,10-Octadecadienoic acid, methyl ester (2.50%), 9,12-octadecadienoic acid (z,z) (1.60%), and 9-Octadecenoic acid (z)-, methyl ester (1.55%), which were the most abundant compounds detected. These compounds were identified in a methanolic extract of *Rhodiola rosea*. In addition, please see the HPLC analysis of *Rhodiola rosea* extract (Figure S1 and Table S1).

### 3.2. Effect of RHO and CARV Alone on Body Weight and Cardiac Injury Markers

Animals in the control, RHO, and CARV groups appeared healthy with a normal body weight, while the TIL-treated group showed a significantly decreased BW. Pretreatment with RHO and CARV (standard) alone increased BW (Table 2). Regarding cardiac injury markers, serum LDH, CK-MB, and cTI levels were significantly increased in the TIL-treated group. At the same time, pretreatment with RHO and CARV alone considerably decreased compared to the TIL-treated group but was still higher than those recorded in normal rats (Table 3).

### 3.3. Impacts of RHO and CARV Alone on TIL-Induced Changes in Inflammatory Cytokines

The TIL-injected group significantly increased the inflammatory IL-1β and TNF-α cytokines. TIL decreased IL-10 levels (Table 3). The pre-administration of RHO and CARV alone to TIL-injected rats showed a protective effect and significant decreases in inflammatory cytokine levels. In contrast, an apparent increase in IL10 levels was reported compared to other groups (Table 4).

### 3.4. Effect of RHO and CARV Alone on Oxidative Stress Biomarkers (ROS, PC, MDA) and TAC in Cardiac Tissue Homogenate

**Figure 2:**
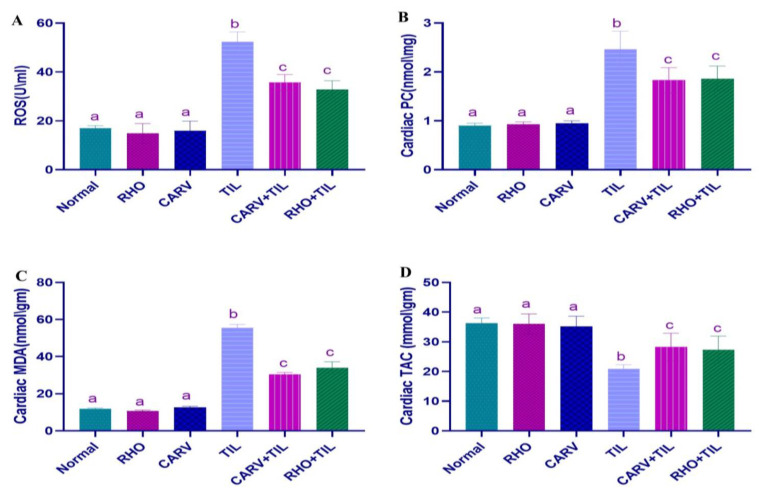
Impacts of Rhodiola against tilmicosin-induced cardiotoxicity on oxidative stress markers. (**A**) ROS, (**B**) Cardiac PC, (**C**). Cardiac MDA, and (**D**) Cardiac TAC. The values represent means ± standard deviations for six separate rats per treatment. ^a,b,c^ Values with different letters are statistically different at *p* < 0.05.

Compared with the control, the oxidative injury markers ROS, PC, and MDA contents in the heart were significantly increased in the TIL-treated rats. At the same time, pretreatment with RHO and CARV alone considerably decreased compared with the TIL-treated group (Figure 2A–C). TAC in TIL-treated rats compared to control was significantly reduced; however, pretreatment of RHO and CARV alone significantly increased the cardiac TAC levels compared to the TIL-treated group (Figure 2D).

In addition, it was shown that the OGG1 protein expression quantity was significantly decreased by the TIL administration in other treated groups. However, the pretreatment of RHO and CARV alone or in combination with the TIL significantly increased the cardiac OGG1 levels compared to the TIL-treated group, and this finding was proved by the OGG1 protein expression using the Western blot analysis. Conversely, the quantitative analysis of the 8-OHdG protein showed that TIL upregulated the expression of 8-OHdG compared with the control, RHO, and CARV groups and downregulated the expression of 8-OHdG. In comparison, the pre-administration of RHO and CARV alone or in combination with TIL protected rats that received TIL, as RHO and CARV significantly increased the OGG1 protein expression and significantly decreased the 8-OHdG protein in relation to the TIL-treated rats. (Please see Supplementary Figure S2.)

### 3.5. Impacts of RHO and CARV Alone against TIL-Induced Alteration in Cardiac mRNA Gene Expression

**Figure 3:**
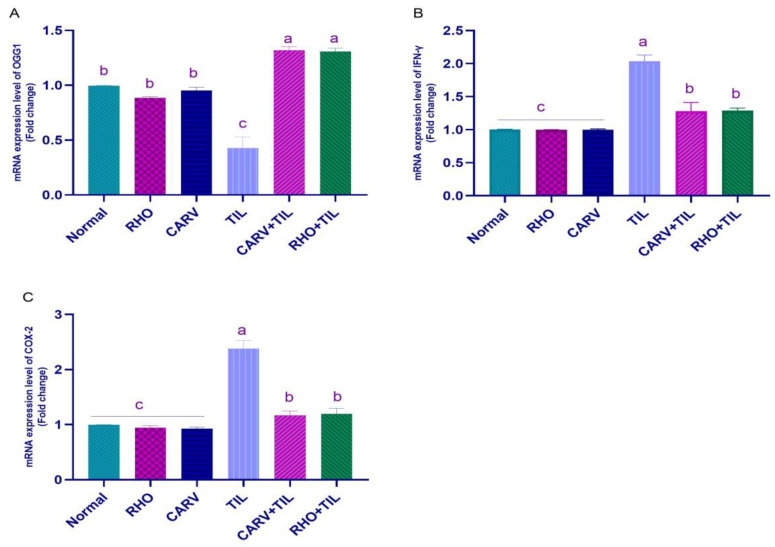
Quantification of mRNA expression of (**A**) OGG1, (**B**) INF-γ, and COX-2 (**C**). Graphic presentations of examined genes were based on qRT-PCR analysis for INF-γ, COX-2, and OGG1 after normalization with GAPDH in different groups. The values represent means ± standard deviations for six separate rats per treatment. ^a,b,c^ Values with different letters are statistically different at *p* < 0.05.

**Figure 4:**
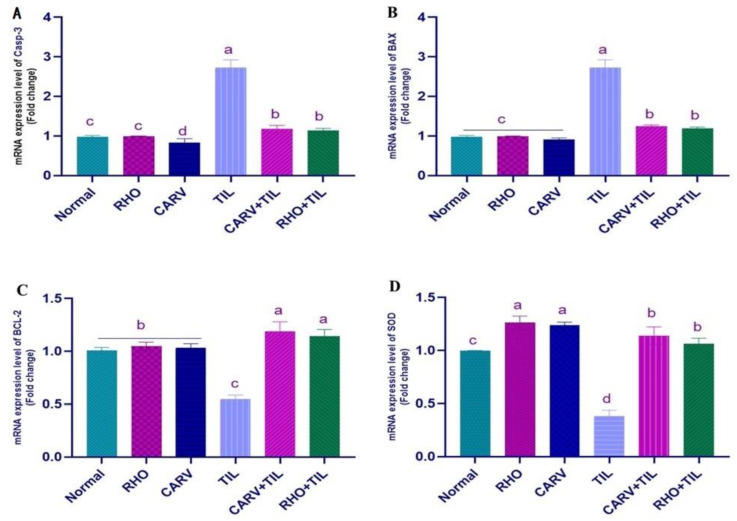
Quantification of mRNA expression of genes. The graphic presentations of examined genes were based on qRT-PCR analysis for (**A**) caspase-3, (**B**) BAX, (**C**) B-cell lymphoma-2 (Bcl-2), and (**D**) superoxide dismutase (SOD) genes after normalization with GAPDH in different groups. The values represent means ± standard deviations for six separate rats per treatment. ^a,b,c,d^ Values with different letters are statistically different at *p* < 0.05.

TIL upregulated the mRNA expression of INF-γ and COX-2 compared with the control, RHO, and CARV groups. In contrast, the pre-administration of RHO and CARV alone in rats protected those that received TIL, as it downregulated the examined genes significantly (Figure 3A–C). TIL upregulated the expression of apoptotic genes caspase-3 and BAX compared with control, RHO, and CARV groups and downregulated the expression of the anti-apoptotic gene BCL-2, antioxidant gene SOD, and OGG1 mRNA expression. In comparison, the pre-administration of RHO and CARV alone protected rats that received TIL, as RHO and CARV significantly decreased the mRNA expression of caspase-3, BAX, INF-γ, and COX-2 while significantly increasing the mRNA expression of BCL-2. SOD and OGG1 resorted to the examined genes (Figure 3A–D and Figure 4A).

### 3.6. Protective Effect of RHO and CARV Alone against TIL-Induced Cardiac Muscle Histopathology

**Figure 5:**
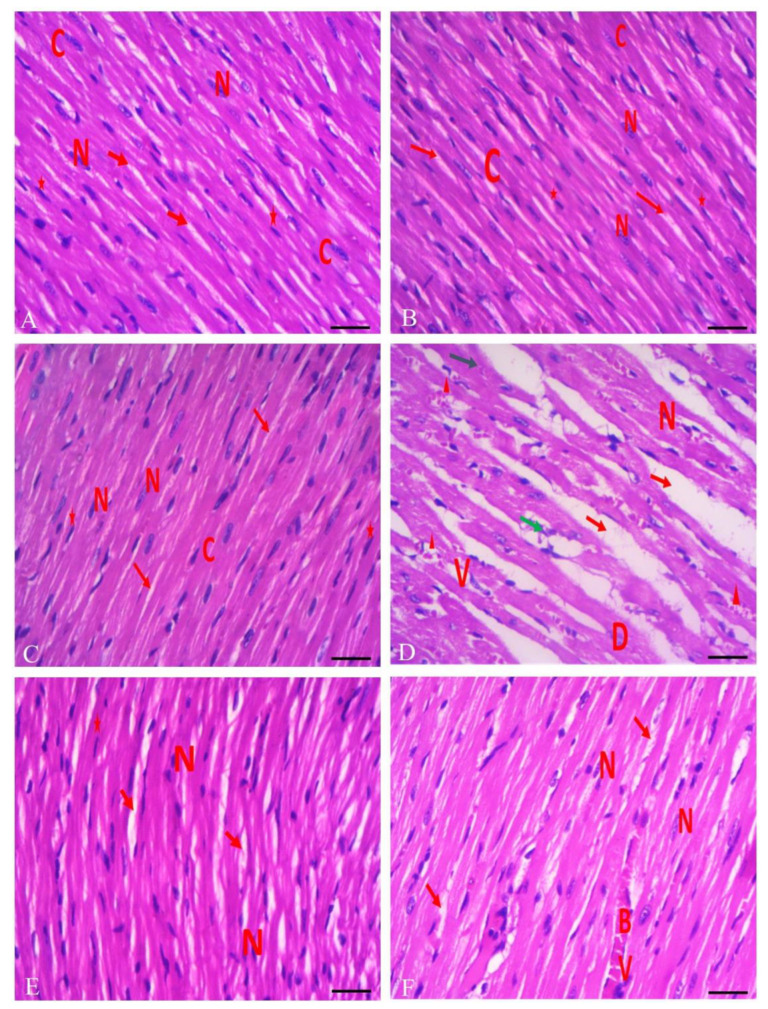
A photomicrograph of a longitudinal section of the myocardium of the left ventricle of control rats (**A**–**C**) showing the normal structure of the myocardium, formed of branching and anastomosing cardiac muscle fibers with centrally located oval nuclei (N) and acidophilic cytoplasm (**C**) and delicate connective tissue endomysium (red arrows) with dark oval nuclei of fibroblasts (star), surrounds the cardiac muscle fibers. (**D**) TIL-treated rats showed loss of the normal architecture of the cardiac muscle fibers with destructed wavy fibers with loss of branching and anastomosing, with some areas showing focal necrosis (green arrow) with intercellular hemorrhage (arrowhead) and multiple dilated congested blood vessels (BV) surrounded by monocellular infiltration (CI) with an increased number of fibroblasts (star), nuclei of cardiomyocytes appearing with small darkly stained nuclei (N), areas of hyaline degeneration (blue arrow), and widening of endomysium. (**E**) CARV + TIL-treated rats and (**F**) RHO + TIL-treated rats showed a normal structure of the myocardium, formed of branching and anastomosing cardiac muscle fibers with centrally located oval nuclei (N) and dark oval nuclei of fibroblasts (star). However, there is still a widened endomysium (red arrow) with congested blood vessels (BV) in the RHO + TIL-treated group (H&E X400). Scale bar = 50 μm.

The myocardium of the left ventricle of control rats showed the typical structure of the myocardium, which is formed of branching and anastomosing cardiac muscle fibers with centrally located oval nuclei and acidophilic cytoplasm, and a delicate connective tissue endomysium with dark oval nuclei of fibroblasts that surrounds the cardiac muscle fibers. On the other hand, TIL-treated rats showed loss of the typical architecture of the cardiac muscle fibers with destructed wavy fibers with loss of branching and anastomosing, with some areas showing focal necrosis with intercellular hemorrhage and multiple dilated congested blood vessels surrounded by monocellular infiltration with an increased number of fibroblasts, nuclei of cardiomyocytes appearing with small darkly stained nuclei, areas of hyaline degeneration, and widening of the endomysium. In contrast, CARV + TIL-treated rats and RHO + TIL-treated rats showed the normal structure of the myocardium, formed of branching and anastomosing cardiac muscle fibers with centrally located oval nuclei and dark oval nuclei of fibroblasts. However, there is a widened endomysium with congested blood vessels in the RHO + TIL-treated group (Figure 5A–F).

### 3.7. Immunohistochemical Analysis of the Protective Effect of RHO and CARV Alone in TIL-Induced Cardiac Muscle

**Figure 6:**
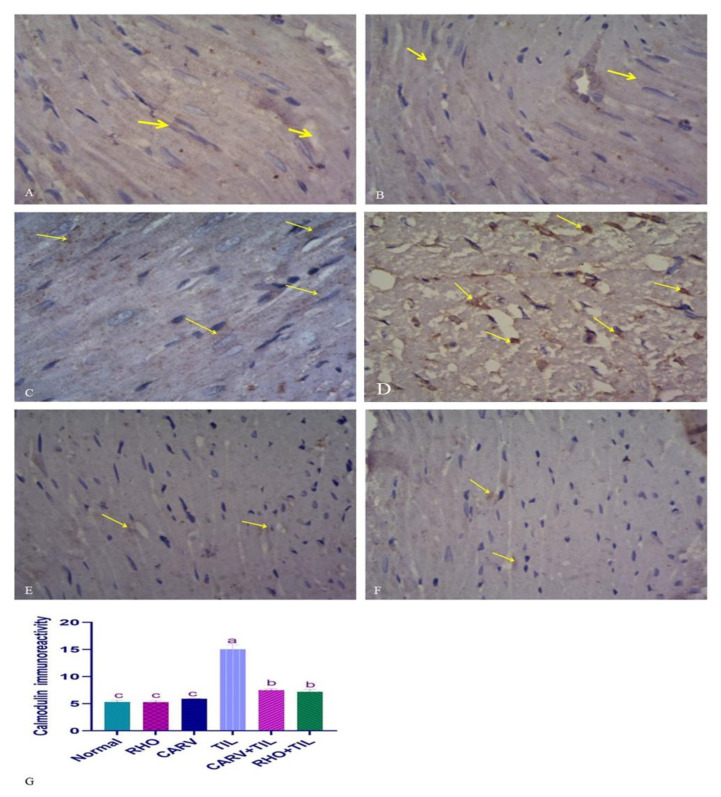
Immunohistochemical staining of calmodulin on the experimental rats’ cardiac cells (IHC, ×1000). (**A**–**C**) Control rats showing normal expression of calmodulin (brown color) (yellow arrow). (**D**) TIL-intoxicated rats showed a marked increase in calmodulin expression in multiple scattered foci of strong immune positivity (yellow arrow). (**E**) CARV + TIL-treated rats and (**F**) RHO + TIL-treated rats showed decreased calmodulin expression (yellow arrow). Scale bar = 50 μm. The level of positive immunoreactivity of calmodulin (**G**) was analyzed using a one-way ANOVA followed by Tukey’s multiple comparisons test. HMGB1 immunohistochemical staining was evaluated in 10 individual sections. All data are presented as means + SD, ^a,b,c^ Values with different letters are statistically different at *p* < 0.05.

**Figure 7:**
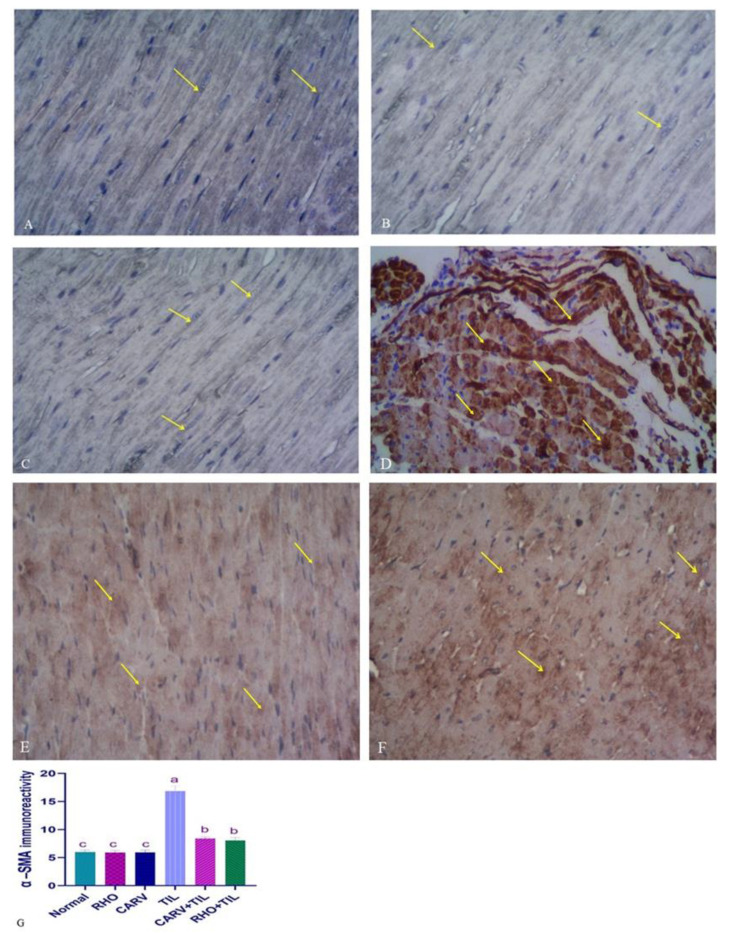
Immunohistochemical staining of alpha-smooth muscle actin (α-SMA) in the experimental rats’ cardiac cells (IHC, ×400). (**A**–**C**) Control rats show normal expression of α-SMA (brown color) (yellow arrow). (**D**) TIL-intoxicated rats showed a marked increase in α-SMA expression in the form of solid immune positivity (yellow arrow). (**E**) CARV + TIL-treated rats and (**F**) RHO + TIL-treated rats showed decreased α-SMA expression (yellow arrow). Scale bar = 50 μm. The level of positive immunoreactivity of α-SMA (**G**) was analyzed using a one-way ANOVA followed by Tukey’s multiple comparisons test. HMGB1 immunohistochemical staining was evaluated in 10 individual sections. All data are presented as means + SD, ^a,b,c^ Values with different letters are statistically different at *p* < 0.05.

For the expression of calmodulin in control, CARV, and RHO rats’ cardiac cells, normal rats show regular expression of calmodulin (brown color). On the contrary, TIL-intoxicated rats showed a marked increase in calmodulin expression in the form of multiple scattered immune solid-positive foci. In contrast, CARV + TIL-treated rats and RHO + TIL-treated rats showed decreased calmodulin expression (Figure 6A–F) for the expression of alpha-smooth muscle actin (α–SMA) in the cardiac tissue of the control, CARV, and RHO rats’ cardiac cells. The control group revealed the normal expression of α–SMA. Conversely, TIL-intoxicated rats showed a marked increase in α–SMA expression in the form of solid immune positivity, while CARV + TIL-treated rats (E) and RHO + TIL-treated rats (F) showed decreased α–SMA expression (Figure 7A–F).

## 4. Discussion

This study confirmed RHO’s protective impacts against TIL-induced cardiac injury. The serum levels of cardiac injury markers, such as lactate dehydrogenase, increased significantly. Cardiac troponin (cTI) and creatine phosphokinase (CK-MB) showed significant weight loss. In addition, genetic, histopathological, and immunohistochemical alterations were observed in the examined hearts of TIL-receiving rodents [4,37]. However, the mechanisms of cardiotoxicity caused by macrolides, including TIL, have not been fully explained in any previously reported trials. One study found that TIL induced adrenaline production, which could worsen circulatory stress and decrease ventricular function [40]. The parameters mentioned above were ameliorated when CARV or RHO was pretreated in TIL-treated rats, in line with others [41].

GC-MS analysis was performed to investigate the chemical constituents of the *Rhodiola rosea* extract. The results revealed significant amounts of various compounds, including n-Hexadecenoic acids (26.47%), á-Sitosterol (5.39%), 2-Propenoic acid, 3-phenyl-, methyl ester (5.11%), Linalool (3.62%), 1,6-Octadien-3-ol, 3,7-dimethyl (3.62%), 7,10-Octadecadienoic acid, methyl ester (2.50%), 9,12-octadecadienoic acid (z,z) (1.60%), and 9-Octadecenoic acid (z)-, methyl ester (1.55%). These compounds are known for their anti-inflammatory and antioxidant properties [42,43,44]. N-hexadecenoic acids, also known as palmitoleic acids, are monounsaturated fatty acids with multiple bodily functions. They exhibit anti-inflammatory properties and play a role in maintaining skin health. In terms of metabolism, n-hexadecenoic acids regulate lipid levels in the body. Additionally, they may improve insulin sensitivity. Furthermore, as signaling molecules, these fatty acids can influence cellular processes such as gene expression, cell proliferation, and differentiation [45,46]. á-Sitosterol, a plant sterol found in plants, has various health benefits. It helps manage cholesterol by reducing its absorption and improving lipid profiles. á-Sitosterol also possesses anti-inflammatory properties. Additionally, á-Sitosterol has immunomodulatory effects, boosting immune function [47]. Linalool possesses anti-inflammatory and analgesic properties. Furthermore, it exhibits antimicrobial activity and antioxidant properties [48].

TIL-induced oxidative tissue damage is associated with elevated ROS, a reduction in the activities of GSH and CAT [49], and DNA damage [37]. Antioxidants are the first line of defense against oxidative stress because they neutralize ROS and other free radicals [50]. The current study showed an increase in protein carbonyl (PC), total reactive oxygen species (ROS), and malondialdehyde (MDA) with an apparent decrease in OGG-1 mRNA expression, total antioxidant capacity (TAC), and SOD mRNA in the cardiac homogenate of TIL-treated rats. Our findings and those of Awad et al. [37] reported that lipid peroxidation and redox dysregulation resulting from TIL have been found to induce cardiac injury. Pre-administration of RHO increased TAC in cardiac homogenate while lowering oxidative stress and DNA damage biomarkers if compared with CARV-pretreated rats (standard). This indicates a crucial function for RHO in controlling cardiac oxidative stress. Previous research [41,51] aligns with ours, documenting that salidroside has an antioxidant effect on cardiac damage.

Damage to the structure and physiology of cells due to oxidative stress can affect things like RNA processing, transcription, translation, the structure and function of the cell membrane, and metabolism [5]. The enhanced ROS generation from TIL-induced cellular damage attracts more inflammatory cells and fibroblasts to the wounded tissue and promotes the release of cytokines like tumor necrosis factor-alpha [5]. TNF-α initiates the induction of other cytokines, such as IL-1β and interferon-γ (IFN-γ) [52]. IFN-γ is a pleiotropic cytokine crucial to pro-apoptotic potentials and immune responses [53]. The commencement of a cascade of pro-inflammatory reactions is the result of a complex interplay between immune cell activity and IFN-γ, requiring the coordinated integration of signals from various pathways involving cytokines and pattern recognition receptors (PRRs) such as interleukins, TNFα, and IFNs [54]. Janus kinases JAK1 and JAK2 are phosphorylated in response to IFN-γ. In response, the activated JAKs phosphorylate the dormant STAT1 (signal transducer and activator of transcription) transcription factor located in the cytoplasm [54]. In our study, TIL treatment significantly showed a rise in TNF-α and IL-1β [55], upregulated IFN-γ and COX-2 mRNA expression in cardiac tissues [55], and a significant decrease in anti-inflammatory IL-10 cytokine [55], leading to an increase in the inflammatory process. In contrast, co-administration of CARV + TIL, or RHO + TIL, resulted in a decrease in serum inflammatory cytokines, IFN-γ, and COX-2 mRNA expression in cardiac tissues with an elevation of IL-10, which can be attributed to the anti-inflammatory effect of RHO. Previous experimental studies reported that RHO has an anti-inflammatory impact on myocardial ischemia by inhibiting the PI3K/Akt/mTOR pathway in vivo and the TLR4/NF-κB signaling pathway [51].

The active compounds contained in *Rhodiola rosea* showed anti-oxidation and anti-inflammation (inhibition in TNF-α, IL-1β, and IL-6) via the MAPK signaling pathway [56]. Salidroside restores mitochondrial structure and function by preventing the buildup of reactive oxygen species. Salidroside’s antioxidant and protective effects come from its ability to selectively inhibit the activation of genes involved in the Mapk7 pathway that induces oxidative stress. These genes include growth arrest and DNA-damage-inducible 45 a (Gadd45a), mitogen-activated protein kinase 7 (Mapk7), and related RAS viral oncogene homolog 2 (Rras2) [57]. Also, salidroside activates the Nrf2 signaling pathway [58].

Changes in Ca^2+^ handling proteins can be attributed to elevated levels of cardiac ROS in human and animal models of cardiac dysfunction. Diastolic dysfunction is exacerbated by Ca^2+^ overload, and Ca^2+^/calmodulin-dependent protein kinase II (CaMKII) changes contribute to this problem [59]. However, oxidative stress and intracellular Ca^2+^ overload, leading to cardiac failure, may be sustained by a vicious loop generated by abnormal Ca^2+^ processing in sick myocytes [60,61]. Reduced cardiac systolic potential and an increase in sudden cardiac mortality may result from excess Ca^2+^ buildup via calcium transporters. The involvement of Ca^2+^/calmodulin-dependent protein kinase II-gamma (CaMKII) in the etiology of cardiotoxicity is primarily attributable to an increase in cytosolic Ca^2+^ levels. Apoptosis is facilitated by CaMKII activation [62]. Here, TIL administration showed significant upregulation of CaMKII expression by IHC, while co-administration of CARV + TIL, or RHO + TIL, decreased its expression. RHO can stabilize the cell membrane’s ion pumps and calcium channels, rebuild the atrial potential in heart failure, inhibit atrial fibrillation, eliminate ectopic rhythm, and reduce atrial fibrosis. Calcium channel proteins in the heart, including myocardial sarcoplasmic reticulum Ca^2+^-ATP Enzyme 2a (SERCA2a), may be responsible for *Rhodiola rosea*’s favorable electrogenic effects [63].

In addition, pro-apoptotic (Bax, caspase-3) and anti-apoptotic (Bcl-2) proteins are significant players in the apoptosis family. Bax activation guarantees cell damage during apoptosis by creating a pore in the mitochondrial membrane. Proteolytic breakdown of cellular components and eventual cell death are triggered by the activation of caspase-3 after the induction of mitochondrial cytochrome-c [32]. Also, the associated proteins, DNA, oxidative stress, and lipid damage have been reported to be strongly linked to the incidence of apoptosis [9]. Our results clarified that TIL caused upregulation of caspase-3, Bax and downregulation of Bcl-2 expression, which reflects the apoptotic role in TIL-induced cardiomyopathy [37]. Also, several studies documented cardio-toxic effects and apoptosis in response to TIL toxicity [64]. RHO’s anti-apoptotic action may help preserve myocardial integrity and reduce myocardial damage because pretreatment with RHO decreased caspase-3 and Bax gene levels while increasing Bcl-2 expression compared to CARV pretreatment. Consistent with prior experimental research and our findings [41,58].

Furthermore, α-smooth muscle actin (α-SMA) is the hallmark of mature myofibroblast expression in cardiac tissues [65]. Numerous cells that resemble fibroblasts are seen in the myocardium. These interstitial cells are quiescent without injury, but various noxious stimuli can trigger cardiac fibroblast activation [66]. Myocardial infarction in humans and animals consistently results in cardiac fibroblasts transforming into α-SMA-positive myofibroblasts [65]. Myofibroblast activation and deactivation are critical for proper tissue repair; nevertheless, prolonged or excessive myofibroblast activity can increase stiffness and cause heart failure [67]. In our work, TIL administration exhibited significant upregulation of α-SMA expression; conversely, pre-administration of CARV + TIL or RHO + TIL showed downregulation of its expression in cardiac tissues by IHC, denoting that RHO has an anti-fibrotic effect. A recent experimental study by Yang et al. [68] reported that SAL significantly reduced α-SMA expression in renal fibrosis. This is in line with our findings.

The p38 MAPK pathway strongly influences myocardial fibrosis. Inflammatory cytokines and other stimuli at the receptor level (TGF-R, TLR4) trigger p38 signaling. These actions will trigger the activation of the upstream activator of the p38 MAPK kinases, mitogen-activated protein kinase MKK3/6. Cross-talk between p38 and IKK-NFkB signaling drives the pro-fibrotic (collagen 1 and 3 and α-SMA) and pro-inflammatory (IL-1, IL-6, and TNF-α) gene programs following p38 activation [66]. Molecular studies of how various cells in the body react to environmental cues should focus on the downstream signaling cascades that regulate transcription. OGG-1 is a DNA glycosylase that removes a mutagenic base after it has been damaged by reactive oxygen species (ROS), and its expression levels can be used to gauge how well RHO protects against the DNA damage caused by Tilmicosin. Oxo guanine-DNA glycosylase-1 (OGG1), a major DNA glycosylase that hydrolyzes oxidized-guanine (8-oxo-dG) to guanine, was also found to be at lower levels during this time. DNA damage was significantly elevated in TIL-induced toxicity, correlated with decreased OGG1 levels. These results thus demonstrated that RHO treatment protects the heart from TIL toxicity by maintaining OGG1 levels and protecting mitochondria from DNA damage; this result was in harmony with [69], in which they showed the maintenance of OGG1 levels and protecting mitochondria from DNA damage. OGG1 is a vital DNA glycosylase enzyme that plays a crucial role in removing a specific type of DNA damage produced by 8-OHdG. This implies that OGG1 protects DNA from damage and reduces the risk of carcinogenesis [70,71]. The findings from our research support the notion that Ogg1 and 8-OHdG are interconnected and provide evidence for the antioxidant abilities of RHO and CARV. Pre-treatment with RHO and TIL reduced the levels of inflammatory cytokines, specifically IFN-γ, COX-2, and mRNA expression in heart tissues. Additionally, it resulted in an increase in IL-10 levels, which could be due to the powerful anti-inflammatory effect of RHO. Previous reports cleared the anti-inflammatory effect of RHO in nonalcoholic fatty liver disease [72,73] and acute lung injury [74]. Also, Abou-Zeid et al. [75] showed that Moringa oleifera possesses anti-inflammatory properties that may shield the kidney from damage caused by Til-induced inflammation. Protein, DNA, and lipid damages leading to apoptosis have been linked to TIL exposure, although the mechanisms behind TIL-induced cardiotoxicity are yet unknown [76]. Furthermore, TIL may deplete antioxidant defense mechanisms in treated animals’ livers, kidneys, and hearts, leading to oxidative stress [77,78,79]. In previous studies [23,24], TIL injections have been demonstrated to result in significant tissue damage via oxidative stress and apoptosis; this knowledge was used to determine the dose employed in the present study [4,28]. When comparing TIL with the control, RHO, and CARV groups, we find that TIL downregulates the expression of the anti-apoptotic gene BCL-2 and the antioxidant gene SOD, while upregulating the expression of the apoptotic gene’s caspase-3 and BAX. Pretreatment with RHO and CARV alone dramatically reversed the studied genes toward resistance to TIL in rats. These results were in harmony with others [66,67,68], confirming the antioxidant, anti-inflammatory, and anti-apoptotic effects of RHO administration. RHO aids in the removal and detoxification of external toxins and plays a crucial function in boosting stressed cells’ antioxidant defense [80].

## 5. Conclusions

The current study has shown that RHO can mitigate the side effects of TIL on the heart. These results demonstrate for the first time that RHO is superior to CARV in preventing cardiac damage caused by TIL toxicity. Cardiac gene expression was also modified, and inflammatory cytokine levels and antioxidant activity in the blood were both affected by TIL toxicity. All biochemical and tissue markers that had been changed were retrieved by pre-administration of RHO. The expression of genes involved in DNA damage, inflammation, antioxidants, and apoptosis was controlled by RHO with downregulation of the inflammatory cytokines’ genes and increased antioxidant and antiapoptotic gene expression. In contrast to CARV-pretreated animals, cellular immunological reactivity to calmodulin and α-SMA was decreased. These findings provided further evidence for using RHO to shield the heart from TIL’s side effects. They opened the door for future research to look for additional signaling pathways involved in such regulation in organs other than the heart.

## 6. Limitations and Future Directions

The study fails to address using an animal model that mimics human cardiac responses in future experiments. Although no animal model can perfectly mimic human cardiac responses, pigs and specific dog breeds are often used in research due to their anatomical and functional similarities to humans. These models provide valuable insights into cardiac physiology and pathologies. It is important to note that while each model has its benefits and limitations regarding their resemblance to human cardiac responses, combining different models and experimental approaches is necessary to understand the complexities of human cardiac responses comprehensively.

The study should incorporate hemodynamic measurements to address the current lack of information. Additionally, further investigations should involve a wide range of age distributions to allow for more accurate disease staging. It would also be valuable to study changes in blood pressure and conduct proteome analysis as practical tools for identifying new proteins associated with various heart diseases and understanding alterations in heart structure.

## Figures and Tables

**Table 1:** Primers for gene expression by RT-PCR.

Gene	Direction	Primer Sequence	Annealing Temp.	(bp)	Accession Number
OGG1	F:	ATCTGTTCTTCCAACAACAAC	58 °C	212	NM_030870
	R:	GCCAGCATAAGGTCCCCACAG			
IFN-γ	F:	TGTCATCGAATCGCACCTGATC	57 °C	185	NM_138880
	R:	GACTCCTTTTCCGCTTCCTTAG			
Bax	F:	GGCGAATTGGCGATGAACTG	57 °C	167	NM_017059.2
	R:	ATGGTTCTGATCAGCTCGGG			
Bcl-2	F:	GATTGTGGCCTTCTTTGAGT	57 °C	172	NM_016993.1
	R:	ATAGTTCCACAAAGGCATCC			
SOD	F:	AGGATTAACTGAAGGCGAGCAT	59 °C	173	NM_017050.1
	R:	TCTACAGTTAGCAGGCCAGCAG			
Caspase-3	F:	TGTCAGCTACTCCCAGGTTG	57 °C	146	NM_012922
	R:	TCAAGAAGGTGGTGAAGCAG			
COX-2	F:	CATGGGAGTTGGGCAGTC	56 °C	70	AF159101
	R:	TCAATCTCGGGTGGCTGAACG			
GAPDH	F:	AGGTGGAAGAATGGGAGTTG	55 °C	197	NM_017008.4
	R:	GCTTTCTCCAACCTCTCCTACTACA			

**Table 2:** GC-MS analysis of *Rhodiola rosea* extract.

No.	Retention Time (min)	(Chemical Compound)	Area%	MF	Molecular Formula	Molecular Weight
1	6.85	Linalool	3.62	797	C10H18O	154
2	6.85	1,6-Octadien-3-ol, 3,7-dimethyl	3.62	833	C10H18O	154
3	8.88	Benzene, 1-methoxy-4-(1-propenyl)-	1.41	784	C10H12O	148
4	8.88	Estragole	1.41	866	C10H12O	148
5	12.65	Guaiacol, 6-propenyl-	1.17	831	C10H12O2	164
6	12.65	Phenol, 2-methoxy-6-(1-propenyl)-	1.17	827	C10H12O2	164
7	13.15	2-Propenoic acid, 3-phenyl-, methyl ester	5.11	897	C10H10O2	162
8	15.08	Trans-à-Bergamotene	1.77	919	C15H24	204
9	16.76	á-Copaene	0.92	839	C15H24	204
10	24.16	Ethanol, 2-(9-octadecenyloxy)-, (Z)-	0.73	776	C20H40O2	312
11	25.62	pentadecanoic acid, 14-methyl-, methyl ester	1.16	798	C17H34O2	270
12	25.62	Palmitic Acid methyl ester	1.16	787	C17H34O2	270
13	25.62	Hexadecanoic acid, methyl ester	1.16	789	C17H34O2	270
14	26.44	n-Hexadecanoic acid	26.47	921	C16H32O2	256
15	28.62	7,10-Octadecadienoic acid, methyl ester	2.50	876	C19H34O2	294
16	28.80	9-Octadecenoic acid (z)-, methyl ester	1.55	877	C19H36O2	296
17	29.53	9-Octadecenoic acid (z)-	0.93	819	C18H34O2	282
18	29.63	Oleic Acid	1.15	781	C18H34O2	282
19	29.63	cis-Vaccenic acid	1.15	792	C18H34O2	282
20	29.63	9-Octadecenoic acid (z)-	1.15	809	C18H34O2	282
21	29.63	trans-13-Octadecenoic acid	1.15	796	C18H34O2	282
22	31.94	Alanine	1.16	665	C14H14F3NO4	317
23	35.79	4H-1-Benzopyran-4-one, 2-(3,4-dihydroxyphenyl)-6,8-di-á-d glucopyranosyl-5,7-dihydroxy	1.04	708	C27H30O16	610
24	40.03	17-Pentatriacontene	1.01	677	C35H70	490
25	41.00	4H-1-Benzopyran-4-one, 2-(3,4-dimethoxyphenyl)-3,5-dihydroxy-7-methoxy	0.99	733	C18H16O7	344
26	41.00	Dasycarpidan-1-methanol, acetate (ester)	0.99	730	C20H26N2O2	320
27	41.91	Oleic acid, eicosyl ester	1.56	667	C38H74O2	562
28	43.04	Isochiapin B	1.60	717	C19H22O6	346
29	43.04	9,12-octadecadienoic acid (z,z)	1.60	731	C27H54O4Si2	498
30	43.89	.Psi.,.Psi.-Carotene, 1,1′,2,2′-tetrahydro-1,1′-dimet hoxy	1.45	672	C42H64O2	600
31	44.72	á-Sitosterol	5.39	786	C29H50O	414
32	45.00	Isochiapin B	3.11	725	C19H22O6	346

**Table 3:** Impacts of RHO and CARV alone against TIL-induced cardiotoxicity on body weight and cardiac injury markers.

	Normal	RHO	CARV	TIL	CARV + TIL	RHO + TIL
B.W (gm)	195.6 ± 3.1 ^a^	199.1 ± 5.1 ^a^	198.0 ± 5.0 ^a^	154.0 ± 4.8 ^c^	170.1 ± 3.5 ^b^	168.6 ± 3.1 ^b^
LDH (U/L)	707.50 ± 12.6 ^b^	721.83 ± 13.2 ^b^	717.16 ± 11.7 ^b^	1427.33 ± 39.2 ^a^	600.16 ± 11.1 ^c^	601.83 ± 10.9 ^c^
CK-MB (U/L)	701.50 ± 13.03 ^b^	697.66 ± 19.38 ^b^	700.33 ± 12.19 ^b^	1205.33 ± 28.71 ^a^	571.16 ± 8.841 ^c^	572.33 ± 8.71 ^c^
Cardiac cTI (Pg/mL)	0.31 ± 0.04 ^c^	0.31 ± 0.05 ^c^	0.32 ± 0.06 ^c^	1.8367 ± 0.27 ^a^	1.060 ± 0.08 ^b^	1.06 ± 0.08 ^b^

The values represent means ± standard deviations for six separate rats per treatment. ^a,b,c^ Values with different letters are statistically different at *p* < 0.05.

**Table 4:** Impacts of RHO and CARV alone on TIL-induced cardiotoxicity on inflammatory and anti-inflammatory cytokines.

	Normal	RHO	CARV	TIL	CARV + TIL	RHO + TIL
TNF (pg/mL)	100.16 ± 2.04 ^c^	105.33 ± 2.16 ^c^	103.00 ± 2.36 ^c^	202.66 ± 6.28 ^a^	160.33 ± 2.92 ^b^	165.02 ± 3.02 ^b^
IL-1 B (pg/mL)	119.16 ± 2.85 ^c^	121.83 ± 2.92 ^c^	120.66 ± 3.07 ^c^	230.5 ± 4.27 ^a^	177.66 ± 5.92 ^b^	176.00 ± 5.79 ^b^
IL-10 (pg/mL)	303.33 ± 5.04 ^a^	306.83 ± 4.83 ^a^	308.33 ± 4.96 ^a^	149.33 ± 2.94 ^c^	208.83 ± 4.35 ^b^	202.33 ± 5.31 ^b^

The values represent means ± standard deviations for six separate rats per treatment. ^a,b,c^ Values with different letters are statistically different at *p* < 0.05.

## Data Availability

Upon request.

## Supplementary Materials

The following supporting information can be downloaded at: https://www.mdpi.com/article/10.3390/toxics11100857/s1. Figure S1: HPLC peaks; Figure S2: Quantification of protein content of (A) Ogg1, (B) 8-OHdG, and (C) Western blotting and densitometric quantification of the protein expression of OGG1 following different treatment; Table S1: Rhodiola Rosea Extract (compound names).
