# Supplementary material for: Exploration of Tilmicosin Cardiotoxicity in Rats and the Protecting Role of the Rhodiola rosea Extract: Potential Roles of Cytokines, Antioxidant, Apoptotic, and Anti-Fibrotic Pathways
Source: Toxics. 2023 Oct 13;11(10):857. doi: 10.3390/toxics11100857 (PMC10610616; doi:10.3390/toxics11100857)
Supplement: Supplementary file 1 [file toxics-11-00857-s001.zip › toxics-2596756-supplementary.pdf]

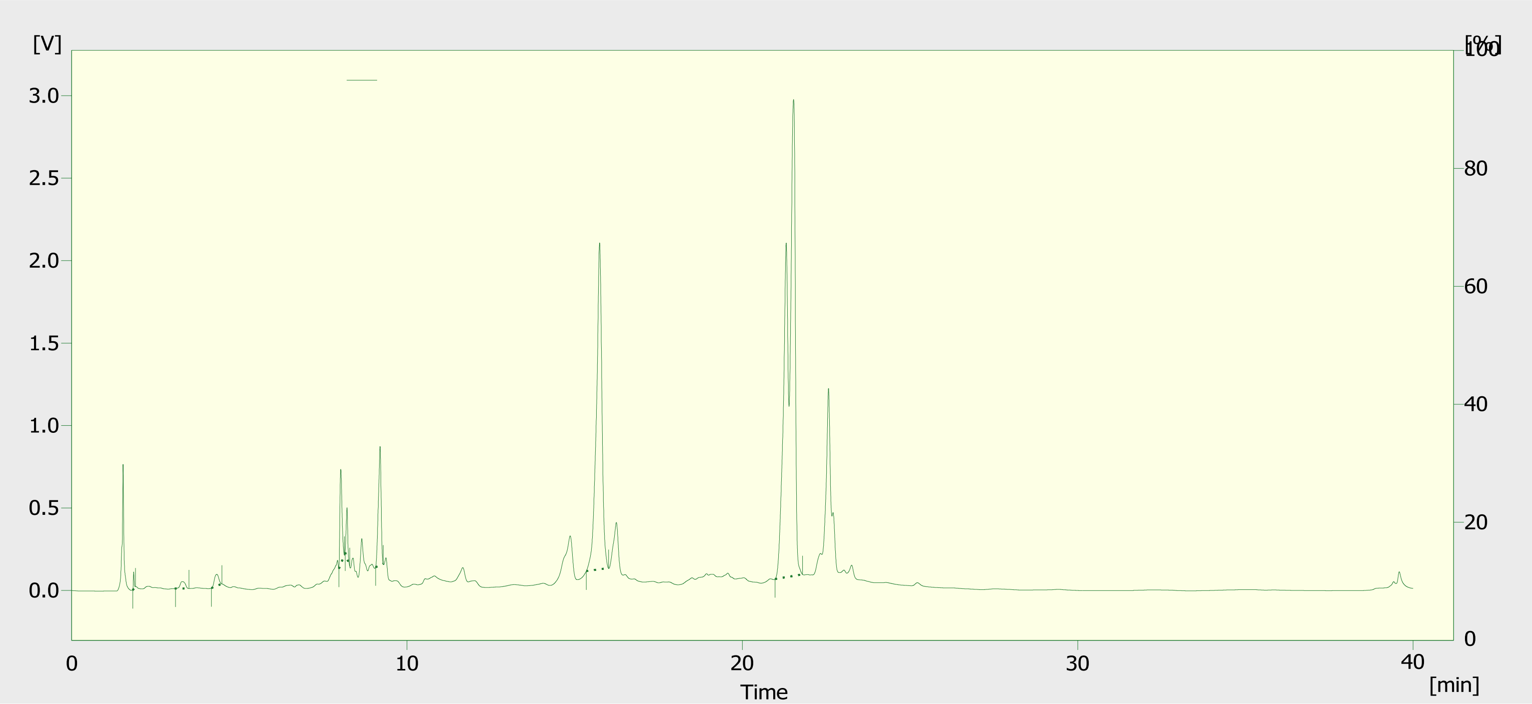

Figure S1. HPLC peaks.

Table S1. Rhodiola Rosea Extract (compound names).

	Signal Name	Reten. Time [min]	Response	Amount [mg/l]	Compound Name
1	Channel 1	1.848	176.123	0.003710	Gallic Acid
2	Channel 1	3.293	450.865	0.000010	Trans Ferulic Acid
3	Channel 1	4.327	646.512	0.002021	Para Amino Benzoic Acid
4	Channel 1	8.023	2493.633	0.105928	Vanillic Acid
5	Channel 1	8.210	1019.886	0.016094	Syringic Acid
6	Channel 1	9.195	4047.686	0.000059	Apeinine
7	Channel 1	15.742	22727.052	2.035400	Rutin
8	Channel 1	21.527	47287.849	27.692959	Sylimarine
		All Signals Total		29.856171	

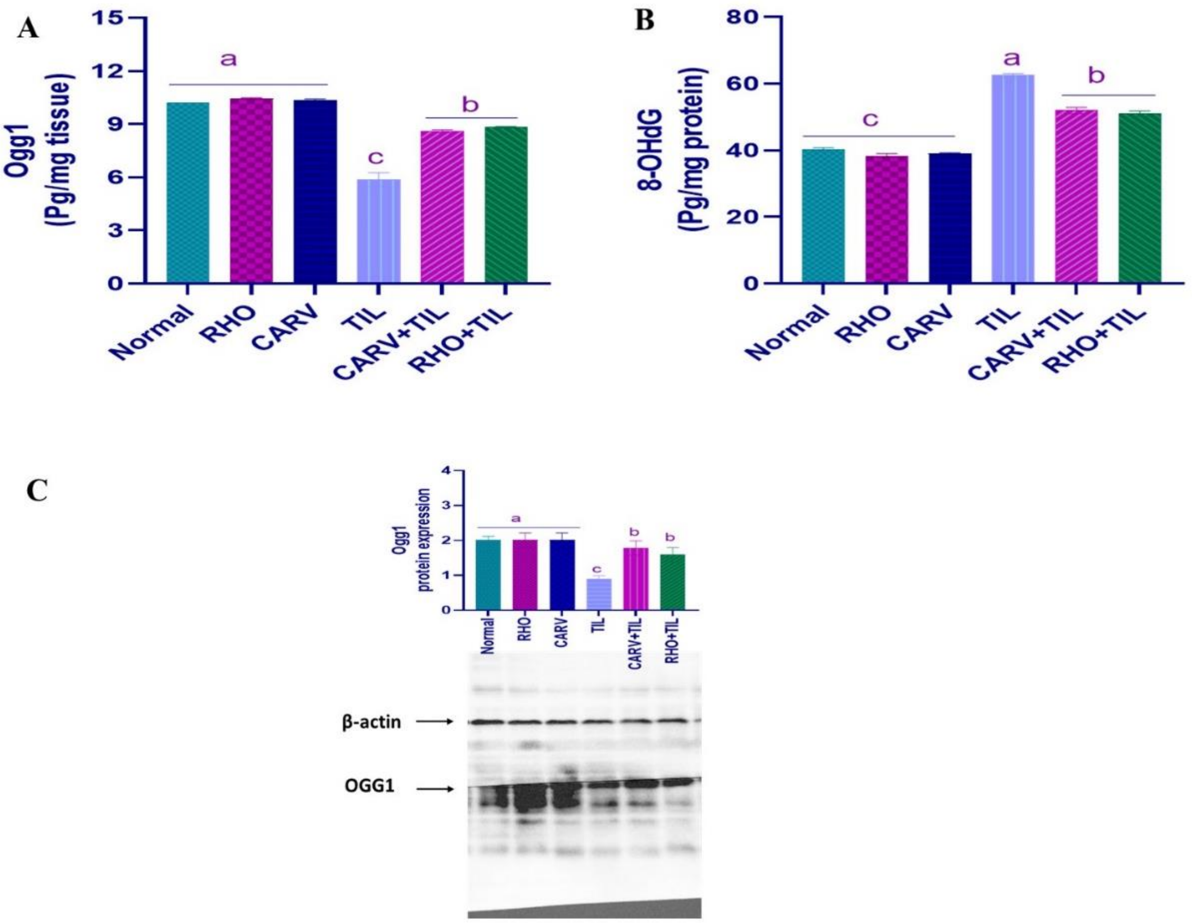

Figure S2. Quantification of protein content of (A) Ogg1, (B) 8-OHdG, and (C) Western blotting and densitometric quantification of the protein expression of OGG1 following different treatment. The values represent means ± standard deviations for six separate rats per treatment. a,b,c Values with different letters are statistically different at $p < 0.05$.
